# Robust BOLD Responses to Faces But Not to Conditioned Threat: Challenging the Amygdala's Reputation in Human Fear and Extinction Learning

**DOI:** 10.1523/JNEUROSCI.0857-21.2021

**Published:** 2021-12-15

**Authors:** Renée M. Visser, Joe Bathelt, H. Steven Scholte, Merel Kindt

**Affiliations:** ^1^Department of Psychology, University of Amsterdam, 1018 WT, Amsterdam, The Netherlands; ^2^Department of Psychology, Royal Holloway University of London, Egham TW20 0EX, United Kingdom

**Keywords:** amygdala, emotional memory, extinction learning, functional magnetic resonance imaging, Pavlovian fear conditioning, translational science

## Abstract

Most of our knowledge about human emotional memory comes from animal research. Based on this work, the amygdala is often labeled the brain's “fear center”, but it is unclear to what degree neural circuitries underlying fear and extinction learning are conserved across species. Neuroimaging studies in humans yield conflicting findings, with many studies failing to show amygdala activation in response to learned threat. Such null findings are often treated as resulting from MRI-specific problems related to measuring deep brain structures. Here we test this assumption in a mega-analysis of three studies on fear acquisition (*n* = 98; 68 female) and extinction learning (*n* = 79; 53 female). The conditioning procedure involved the presentation of two pictures of faces and two pictures of houses: one of each pair was followed by an electric shock [a conditioned stimulus (CS^+^)], the other one was never followed by a shock (CS^–^), and participants were instructed to learn these contingencies. Results revealed widespread responses to the CS^+^ compared with the CS^–^ in the fear network, including anterior insula, midcingulate cortex, thalamus, and bed nucleus of the stria terminalis, but not the amygdala, which actually responded stronger to the CS^–^. Results were independent of spatial smoothing, and of individual differences in trait anxiety and conditioned pupil responses. In contrast, robust amygdala activation distinguished faces from houses, refuting the idea that a poor signal could account for the absence of effects. Moving forward, we suggest that, apart from imaging larger samples at higher resolution, alternative statistical approaches may be used to identify cross-species similarities in fear and extinction learning.

**SIGNIFICANCE STATEMENT** The science of emotional memory provides the foundation of numerous theories on psychopathology, including stress and anxiety disorders. This field relies heavily on animal research, which suggests a central role of the amygdala in fear learning and memory. However, this finding is not strongly corroborated by neuroimaging evidence in humans, and null findings are too easily explained away by methodological limitations inherent to imaging deep brain structures. In a large nonclinical sample, we find widespread BOLD activation in response to learned fear, but not in the amygdala. A poor signal could not account for the absence of effects. While these findings do not disprove the involvement of the amygdala in human fear learning, they challenge its typical portrayals and illustrate the complexities of translational science.

## Introduction

The science of emotional memory provides the foundation of numerous theories on psychopathology, spanning studies in rodents and studies in patients with stress and anxiety disorders. The success of translating mechanistic insights from nonhuman animals to human dysfunction/function depends on careful translation between levels of increasing complexity (and back), and critically evaluating the appropriateness of such translation. The typical paradigm to study fear learning and memory is Pavlovian conditioning. In this model, a neutral stimulus [i.e., a conditioned stimulus (CS^+^); e.g., a geometric shape] is paired with an aversive outcome [unconditioned stimulus (US); e.g., electric shock], by which the neutral stimulus acquires an aversive association and elicits a fear response. The appeal of this model is its robustness (effects are replicable), and the availability of behavioral, physiological, and psychological readouts of the learning process, making it ideal for research across species (Fanselow and Pennington, 2018; Haaker et al., 2019).

Based on work in nonhuman animals the amygdala has obtained the reputation of being a hub in the brain's fear circuitry, in the media regularly referred to as the brain's “fear center” (critically discussed by LeDoux, 2020). Although it is unknown whether defensive responses in animals entail a subjective experience of fear (LeDoux, 2014), mechanistic work in rodents and nonhuman primates directly informs theories on human fear learning (Haaker et al., 2019). Decades of work in animals have shown that the amygdala has a critical role in the acquisition, storage, and/or expression of CS–US associations, as well as in creating fear-inhibiting memory traces as a result of extinction learning (LeDoux, 2000; Davis et al., 2008; Tovote et al., 2015). It also highlights that the amygdala is not a singular, indivisible natural kind, but a collection of structurally and functionally diverse subnuclei (Swanson and Petrovich, 1998).

It is, however, not self-evident that the neural circuitry underlying emotional learning and memory is conserved across species. While selective damage to the amygdalar complex in humans is associated with weaker fear conditioning (Bechara et al., 1995; Klumpers et al., 2015), functional magnetic resonance imaging (fMRI) studies have yielded conflicting findings. Whereas some report amygdala responses to learned threat (CS^+^ > CS^–^; Büchel et al., 1998; LaBar et al., 1998; Reddan et al., 2018; Sjouwerman et al., 2020), recent influential meta-analyses on threat conditioning (Fullana et al., 2016) and extinction (Fullana et al., 2018) did not reveal amygdala activation at all. Explanations for conflicting findings can be summarized as follows: (1) insufficiently fear-provoking conditioning procedures; (2) heterogeneity of experimental designs and analytical methods; and (3) the functional heterogeneity of the amygdala (subtle effects) combined with small study samples leading to a lack of statistical power (Sehlmeyer et al., 2009; Mechias et al., 2010; Fullana et al., 2016, 2018, 2020; Sevenster et al., 2018; Shackman and Fox, 2021).

Here we focus on a key alternative interpretation. The amygdala is located deep within the temporal lobe near cavities, causing dropout of BOLD signal (Weiskopf et al., 2006; Olman et al., 2009). Therefore, one of the prevailing *post hoc* explanations for null findings is failure to measure a proper signal in this area (Sehlmeyer et al., 2009; Fullana et al., 2020). This implies that with a proper signal, amygdala responses to learned threat should be observed. Here, we test this assumption by directly assessing susceptibility artifacts, and by capitalizing on the known responsiveness of the amygdala to faces (Rutishauser et al., 2015), in a mega-analysis of three independent fear-conditioning studies (Visser et al., 2011, 2013, 2015; total, *n* = 98). The experimental paradigm involved the repeated presentation of two pictures of neutral faces and two pictures of houses. During acquisition, one of each pair was followed by an electric shock (CS^+^; 46% reinforcement), while the other one was never reinforced (CS^–^). During extinction learning, none of the stimuli were reinforced. This large dataset offers the unique opportunity to examine amygdala activation to learned threat (CS^+^ vs CS^–^) and to test, using the exact same data (contrasting faces with houses), whether a poor signal could account for an absence of effects. We also explored the role of the bed nucleus of the stria terminalis (BNST), a key subdivision of the central extended amygdala that has been implicated in fear and anxiety.

## Materials and Methods

### Participants

In the current study, a total of 98 complete fear acquisition datasets from healthy volunteers (68 female, 30 male) between 18 and 33 years of age are included (Table 1), with a subsample of 79 participants (53 female, 26 male) also having data on extinction learning. This already excludes some participants (study 1, *n* = 3; study 2, *n* = 16; study 3, *n* = 11) based on criteria that have been detailed previously (Visser et al., 2011, 2013, 2015), such as excessive head motion, sleepiness, equipment failure, and unawareness of stimulus–outcome contingencies. Participants earned partial course credit or financial reimbursement for their participation. All participants gave their written informed consent before participation, were screened to have normal or corrected-to-normal vision, and were naive to the purpose of the experiment. Procedures were executed in compliance with relevant laws and institutional guidelines and were approved by the University of Amsterdam's ethics committee (2010-CP-1246, 2011-CP-1565, and 2013-CP-2387).

**Table 1.** Overview of participant characteristics per study

	*n*	Sex (M/F)	Age (years)		STAI-T		ASI		US intensity (mA)		US unpleasantness	
			Mean (SD)	Range	Mean (SD)	Range	Mean (SD)	Range	Mean (SD)	Range	Mean (SD)	Range
Study 1	19	4/15	22.1 (3.3)	18–31	36.5 (11.4)	22–59	10.3 (5.6)	3–29	19.6 (5.0)	11–32	NA	NA
Study 2	38	15/23	23.7 (3.8)	18–33	35.0 (8.6)	20–52	9.5 (5.5)	2–21	34.6 (15.6)	8–71	3.4 (1.3)	1.5–7
Study 3	41	11/30	20.6 (1.8)	18–24	34.7 (8.8)	22–53	10.6 (5.2)	2–23	26.5 (13.3)	8–60	3.6 (1.5)	1.5–8
Total	98	30/68	22.1 (3.3)	18–33	35.2 (9.2)	20–59	10.1 (5.4)	2–29	28.3 (14.2)	8–71	3.5 (1.4)	1.5–8

Study 1 is Visser et al (2011), Study 2 is Visser et al. (2013), and Study 3 is Visser et al. (2015). US unpleasantness was measured on a scale from 1 (extremely unpleasant) to 9 (not unpleasant). NA, Not available; M, male; F, female; ASI, Anxiety Status Inventory.

### Experimental design

The conditioning procedure was similar across studies and took place during fMRI scanning. It involved the presentation of two CS^+^, a picture of a face (neutral expression) and a picture of a house, which were followed by electric stimulation (US) delivered to the shin, on 6 of 13 trials (46% reinforcement). The electrical stimulation was delivered twice for 2 ms, with a delay of 300 ms (the second coterminating with the CS), by a current stimulator (model DS7A, Digitimer) through MRI-compatible carbon electrodes attached to the right shinbone. The US intensity was determined by adapting the level individually to be clearly aversive (i.e., participants were encouraged to select the maximum tolerable intensity). Two other stimuli (CSs), also a picture of a face (neutral expression) and a picture of a house, were never followed by electric stimuli. Study 2 had an additional stimulus pair that was followed by neutral sounds; responses to these stimuli are not analyzed here. CSs were presented for 6 s with an interval of 22 s (study 1), or 4 s with an interstimulus interval of 20 s (studies 2 and 3). Each trial onset was triggered by the start of the acquisition of a BOLD-MRI volume. The order of stimulus presentation was fixed (counterbalanced across participants) and consisted of a repeating sequence of four target trials, with filler trials of the same stimuli in between. This target and filler structure was used (1) to control for temporal proximity when comparing trial-to-trial pattern similarity across conditions (although not relevant for the present study, it is necessary to control for this when using similarity analyses; Mumford et al., 2014; Visser et al., 2016); (2) to ensure that shock-related activity could not confound CS-related activity (Büchel et al., 1998); and (3) to make the trial order appear random to the participant. In total, the conditioning phase consisted of 52 trials: 28 target trials (7 per stimulus type) and 24 filler trials (6 per stimulus type), including all reinforced CS^+^ trials (i.e., trials that coterminated with a shock). The first two sequences of target trials (eight trials in total, two per stimulus type) were uninterrupted by (reinforced) filler trials, acting as a baseline/habituation phase.

Two studies had an extinction learning session, either 48 h (study 3) or several weeks (study 2) after fear acquisition. The extinction phase is particularly relevant for translational research given that many of the hypotheses coming from work in nonhuman animals are about (changing) the long-term storage and expression of consolidated fear memories, with implications for persistent fears and treatment for patients. During this session, the shock intensity was explicitly set at the individual level, as determined in the previous session, but none of the CS stimuli were reinforced. The extinction phase also consisted of 52 trials: 28 target trials (7 per stimulus type) and 24 filler trials (6 per stimulus type). For each phase, we constrained our analyses to target trials. The rationale for using this design with target and filler trials has been comprehensively described and tested (Visser et al., 2016).

Participants were told that two of the stimuli might be followed by the electric stimulation, whereas the other stimuli would never be followed by the electric stimulation. They were instructed to learn and remember the specific contingencies. Note that while this is different from “instructed fear learning” (Phelps et al., 2001; Mechias et al., 2010; Klumpers et al., 2017; exp. 2) where participants are told beforehand which stimulus is or is not followed by a shock, our instructions do reduce uncertainty related to the safe stimuli (CS^–^) once contingencies have been learned. This presumably leads to enhanced differential fear responding, compared with paradigms where no instructions are given (Reddan et al., 2018; Sjouwerman et al., 2020), and to fewer unaware participants, though in both protocols direct experience of the shock is central to learning the contingencies. Before and after scanning, participants filled out questionnaires to measure trait and state anxiety (Spielberger et al., 1983) and anxiety sensitivity (Peterson and Reiss, 1992). Apart from BOLD activity, studies 2 and 3 included additional measures of conditioned fear, such as retrospective US expectancies [“How much did you expect a shock when this picture was presented?” from 1 (“certainly not”) to 9 (“certainly”)], US unpleasantness ratings [“How unpleasant was the shock?” from 1 (“extremely unpleasant”) to 9 (“not unpleasant”)] and pupil dilation to the CS (see section Conditioned pupil response). Study 3 was originally set up as a between-subject design, testing the effects of the administration of 20 mg of yohimbine HCl versus placebo on fear acquisition. Given that none of the outcome measures showed any group differences (Visser et al., 2015), we report summary statistics for the entire study sample rather than per group. Notably, similar results were obtained when adding pharmacological manipulation as a fixed factor in the models used to examine voxel-wise amygdala responses to learned threat. Parametric maps for this control analysis are available on OSF (https://osf.io/cq5zr/).

### Conditioned pupil response

In studies 2 and 3 (*n* = 79), pupil dilation responses were collected as an independent measure of anticipatory autonomic arousal (Finke et al., 2021). Pupil size was recorded continuously throughout MRI scanning, using a remote nonferromagnetic infrared long range mount eye tracker (model EyeLink 1000, SR Research). Before task onset, a 9-point calibration procedure was performed. Participants were instructed not to move their eyes and fixate on the center of the screen for as long as a stimulus was presented. Before stimulus onset, a white fixation cross turned pink for 1 s to enable the participant to focus in time.

Eye-tracking data were processed and analyzed in MATLAB (version 2015a; MathWorks). Data were sampled at/downsampled to 500 or 250 Hz. Samples around series of missing samples were regarded as unreliable and were removed (100 ms before and 100 ms after each series of 10 missing samples) and replaced by a linear trend at point, using the entire time series. Trials that experienced substantial signal loss, affecting >50% of the samples of the prestimulus baseline and/or the 4 s after stimulus onset, were treated as a “missing trial” and replaced entirely by estimating the linear trend at point over trials for each condition separately (Visser et al., 2013, 2015, 2016; Leuchs et al., 2019). Participants who ended up having a third or more missing trials per phase were excluded (*n* = 3 for acquisition; *n* = 2 for extinction learning). Next, the interpolated pupil time series was low-pass filtered (third-order Butterworth filter, 4 Hz). The baseline pupil diameter was the average value during the 500 ms before each CS onset. The pupil response to the CS was calculated as the peak change from baseline in a window from 0 to 4 s after picture onset. Next, data were *z*-transformed across all trials within an individual (separately for acquisition and extinction) to reduce between-subject variability.

### fMRI data and image acquisition

All scans were acquired on a 3 T MRI scanner (Achieva TX, Philips). Study 1 used an 8-channel head coil, studies 2 and 3 used a 32-channel head coil. All scans were acquired using gradient echo, echoplanar pulse sequences and covered the whole brain. High-resolution anatomical images were acquired as part of all studies and were used for image registration in the current study (Table 2, sequence details). Foam pads were used in all studies to minimize head motion.

**Table 2.** Overview of scan parameters

Parameter	Study 1	Study 2	Study 3
BOLD fMRI			
Slice orientation	Sagittal	Sagittal	Axial
TR	2000 ms	2000 ms	2000 ms
TE	27.63 ms	27.63 ms	27.63 ms
Flip angle	90°	76.1°	76.1°
Voxel size	2.4 × 2.4 × 3.1 mm	3 × 3 × 3.3 mm	3 × 3 × 3.3 mm
Anatomical T1			
TR	8.141 ms	8.124 ms	8.11 ms
TE	3.74 ms	3.72 ms	3.73 ms
Flip angle	8°	8°	8°

Study 1 is Visser et al. (2011), Study 2 is Visser et al. (2013), Study 3 is Visser et al. (2015). TR, Repetition time; TE, echo time.

### fMRI data preprocessing

All images were converted from native PAR/REC to NIfTI-1 format using the dicm2nii toolbox (https://github.com/xiangruili/dicm2nii). The T1-weighted (T1w) image was corrected for intensity nonuniformity using *N4BiasFieldCorrection* [antsApplyTransforms (ANTs) 2.3.3] and was used as T1w reference throughout the workflow. The T1w reference was then skull stripped with a *Nipype* implementation of the *antsBrainExtraction.sh* workflow (ANTs 2.3.3), using OASIS30ANTs as the target template. Brain tissue segmentation of CSF, white matter (WM) and gray matter (GM) was performed on the brain-extracted T1w using FAST (Zhang et al., 2001; FSL 5.0.9; RRID:SCR_002823). Brain surfaces were reconstructed using recon-all (Dale et al., 1999; FreeSurfer 6.0.1; RRID:SCR_001847), and the brain mask estimated previously was refined with a custom variation of the method to reconcile ANTs-derived and FreeSurfer-derived segmentations of the cortical GM of Mindboggle (RRID:SCR_002438; Klein et al., 2017). Volume-based spatial normalization to the FSL MNI ICBM 152 nonlinear sixth Generation Asymmetric Average Brain Stereotaxic Registration Model standard space (TemplateFlow ID: MNI152NLin6Asym; RRID:SCR_002823; Evans et al., 2012) was performed through nonlinear registration with *antsRegistration* (Avants et al., 2008; ANTs 2.2.0; RRID:SCR_004757) using brain-extracted versions of both T1w volume and template.

For each BOLD run per subject (across all sessions), the following preprocessing was performed. First, a reference volume and its skull-stripped version were generated using a custom methodology of *fMRIPrep*. Head-motion parameters with respect to the BOLD reference (transformation matrices, and six corresponding rotation and translation parameters) are estimated before any spatiotemporal filtering using *mcflirt* (FSL, version 5.0.9; Jenkinson et al., 2002). Susceptibility distortion correction (SDC) was omitted. The BOLD time series (slice-timing correction was not applied) were resampled onto their original, native space by applying the transforms to correct for head motion. These resampled BOLD time series will be referred to as “preprocessed BOLD in original space” or just “preprocessed BOLD.” The BOLD reference was then coregistered to the T1w reference using *bbregister* (FreeSurfer), which implements boundary-based registration (Greve and Fischl, 2009). Coregistration was configured with 6 degrees of freedom. Several confounding time series were calculated based on the preprocessed BOLD, as follows: framewise displacement (FD), spatial standard deviation of successive difference images (DVARS), and three region-wise global signals. FD was computed using two formulations following the studies by Power et al. (2014; absolute sum of relative motions) and Jenkinson et al. (2002; relative root mean square displacement between affines). FD and DVARS are calculated for each functional run, both using their implementations in Nipype (following the definitions by Power et al., 2014). The three global signals are extracted within the CSF, the WM, and the whole-brain masks.

The BOLD time series were resampled into several standard spaces, correspondingly generating the following spatially normalized, preprocessed BOLD runs: MNI152NLin6Asym and MNI152NLin2009cAsym. First, a reference volume and its skull-stripped version were generated using a custom methodology of fMRIPrep. All resamplings can be performed with a single interpolation step by composing all the pertinent transformations (i.e., head motion transform matrices, SDC when available, and coregistrations to anatomical and output spaces). Gridded (volumetric) resamplings were performed using ANTs, configured with Lanczos interpolation to minimize the smoothing effects of other kernels (Lanczos, 1964). Nongridded (surface) resamplings were performed using mri_vol2surf (FreeSurfer). The statistical analyses were performed in the FSL MNI152NLin6Asym space at 2 mm isotropic resolution.

Many internal operations of *fMRIPrep* use Nilearn 0.6.2 (Abraham et al., 2014; RRID:SCR_001362), mostly within the functional processing workflow. For more details of the pipeline, see the section corresponding to workflows in fMRIPrep's documentation (https://fmriprep.org/en/stable/workflows.html).

The statistical analysis presented here included the canonical six motion parameters (i.e., rotation and translation in three directions), and their first temporal derivative as nuisance regressors (Siegel et al., 2014). Importantly, as the degree of spatial smoothing may have an impact on the results, we compared the results using full-width at half-maximum Gaussian smoothing kernels with a diameter of 2, 5, or 8 mm, with the latter two sizes being the most common in fMRI studies on conditioning.

All of the raw imaging data in BIDS format (Gorgolewski et al., 2016) and analysis scripts have been made publicly available in the following online repositories: raw data Study 1, 10.18 112/openneuro.ds003553.v1.0.0; raw data Study 2, 10.18 112/openneuro.ds003550.v1.0.1; raw data Study 3, 10.18 112/openneuro.ds003554.v1.0.0; and derivatives and code for all three studies, https://osf.io/cq5zr/.

### Statistical analysis

#### Conditioned pupil response

The *z*-transformed pupil dilation responses were averaged over face and house stimuli. This was because we were not interested in the difference between faces and houses, as this analysis only served as an independent check for whether the fear-conditioning procedure was successful and to identify “learners” (see section Relation between amygdala and indices of fear). Statistical comparisons of the learned associations were performed by within-subject ANOVA, using SPSS (version 26; IBM). Differential aversive learning and extinction learning were assessed by a main effect of stimulus type (CS^+^ vs CS^–^, averaged over faces and houses) and the interaction of trial (13) × stimulus type (CS^+^ vs CS^–^), tested separately for the acquisition and extinction phase.

#### ROI analysis

##### Anatomical mask definition.

Amygdala masks were obtained from the probabilistic Harvard-Oxford subcortical atlas, created using different probability thresholds (*p* > 0.01, *p* > 0.25, and *p* > 0.50) and binarized. The more conservative thresholds of *p* > 0.25 and *p* > 0.50 allow for inferences with higher anatomical specificity, while results at a liberal threshold are reported to minimize the chance of missing small clusters of activation in the periamygdaloid cortex (see Fig. 2). Furthermore, we explored activation in the BNST, a small region surrounding the internal capsule. While often overlooked, accumulating evidence suggests that this region is consistently involved in conditioned responses to the CS^+^ (Fullana et al., 2016; Klumpers et al., 2017), and to threat anticipation more broadly (Hur et al., 2020), even forming a functional unit with a dorsal region of the central amygdala, together referred to as the “central extended amygdala” (Davis et al., 2010; Shackman and Fox, 2016; Torrisi et al., 2018; Fox and Shackman, 2019; Hur et al., 2020). Research in rodents and nonhuman primates suggests that the extended amygdala has a critical role in threat anticipation, both when the threat is unexpected (e.g., in paradigms modeling general anxiety) and when it is expected (e.g., in Pavlovian fear conditioning; Shackman and Fox, 2016; Fox and Shackman, 2019). Several anatomical BNST masks are available via open source platforms. We used one from Torrisi et al. (2018; https://afni.nimh.nih.gov/afni/community/board/read.php?1,149436,149436).

##### Voxel-wise ROI analysis.

We investigated the voxel-wise BOLD responses within bilateral amygdala regions of interest (ROIs) to ensure detection of smaller effects that would not survive multiple-comparison correction across the whole brain. After preprocessing (including 2, 5, or 8 mm smoothing), imaging data were further analyzed using FSL FEAT version 6.0 software. Four regressors of interest (CS^+^ face, CS^–^ face, CS^+^ house, CS^–^ house) were included in a voxel-wise ROI analysis using a general linear model (GLM), with each of the regressors including seven (target) trials (see subsection Experimental design). Stimulus onsets were modeled using a double gamma hemodynamic response function and a duration of 6 s (study 1) or 4 s (study 2 and 3). Regressors of no interest included filler trials, the USs, temporal derivatives for each regressor, and six motion parameters and their temporal derivatives. Normalized parameter estimates in each contrast of interest (cope) based on first-level analysis were combined across individuals to obtain group-level statistical maps. To this end, we performed a permutation-based analysis with 5000 permutations and threshold-free cluster enhancement (TFCE, Winkler et al., 2014) as implemented in FSL *randomize*. This voxel-wise analysis was conducted separately for the left and right amygdala masks to maximize the chance of detecting even small clusters of activation.

##### Susceptibility artifacts.

We examined potential signal dropout because of field inhomogeneities in anatomically defined amygdala masks (at a liberal threshold of *p* > 0.01) for each individual. Susceptibility artifacts were defined as a drop-off in signal intensity to <50% of the mean EPI signal (acquisition and extinction phase analyzed separately) for that participant (Olman et al., 2009). Dropout was low in the left (mean = 1.7%, SD = 2.1) and right (mean = 1.2%, SD = 1.7) amygdala (range, 0–11.7%). Signal dropout across participants is depicted for the fear acquisition and extinction phase separately (see Fig. 2).

##### Mean percentage signal change.

In addition to the voxel-wise analyses, we averaged activity across voxels in the amygdala mask (at a liberal threshold of *p* > 0.01), per condition (CS^+^ face, CS^–^ face, CS^+^ house, CS^–^ house), per individual. Using SPSS (version 26; IBM), we examined the main effects of picture type (face/house), main effects of learned threat (CS^+^/CS^–^), and an interaction of picture type × learned threat. In addition, we used a paired *t* test to compare the average difference between face and house stimuli, averaged across CS type, to the average difference between CS^+^ and CS^–^, averaged across picture type, to directly compare effects in both contrasts.

##### Whole-brain analysis.

In addition to the ROI analysis, a voxel-wise whole-brain analysis was performed on data with moderate (5 mm) spatial smoothing. Regressors were modeled in a GLM as described in subsection Voxel-wise ROI analysis. We performed a permutation-based analysis with 5000 permutations and TFCE (Winkler et al., 2014), as implemented in FSL *randomize* across the whole brain.

## Results

### Conditioned pupil response

**Figure 1.**
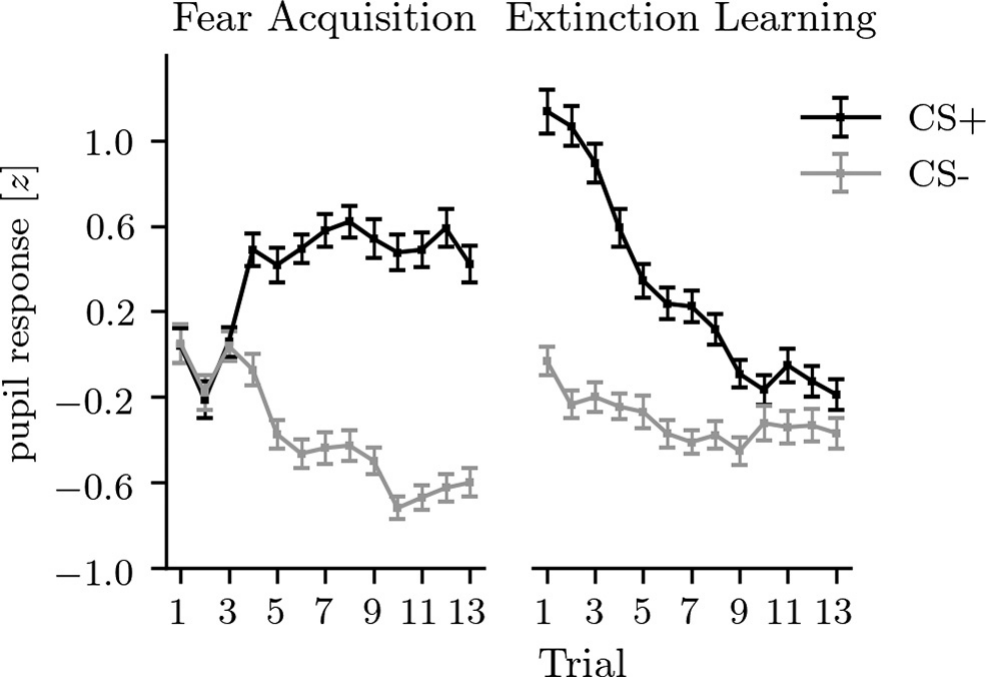
The *z*-transformed pupil dilation responses (peak minus baseline) for the fear acquisition phase (*n* = 76) and the extinction learning phase (*n* = 77) show strong acquisition and extinction of fear. Error bars represent 1 SEM.

Pupil dilation responses were assessed as an independent measure of anticipatory arousal to verify that aversive conditioning was successful. As can be seen in Figure 1, fear acquisition was evident from a trial-by-trial increase in pupil dilation in response to the CS^+^, relative to the CS^–^ (*F*_(12,900)_ = 23.59, *p* < 0.001, η_P_^2^ = 0.24), as well as a main effect of stimulus (*F*_(1,75)_ = 262.47, *p* < 0.001, η_P_^2^ = 0.78). During the extinction learning phase, average responses to the CS^+^ were higher than to the CS^–^ (*F*_(1,76)_ = 129.23, *p* < 0.001, η_P_^2^ = 0.63), and extinction of fear was evident from a decrease in pupil dilation in response to the CS^+^, relative to the CS^–^ (*F*_(12,912)_ = 15.56, *p* < 0.001, η_P_^2^ = 0.17). This indicated that the fear acquisition and extinction learning procedures were successful.

### ROI results

**Figure 2.**
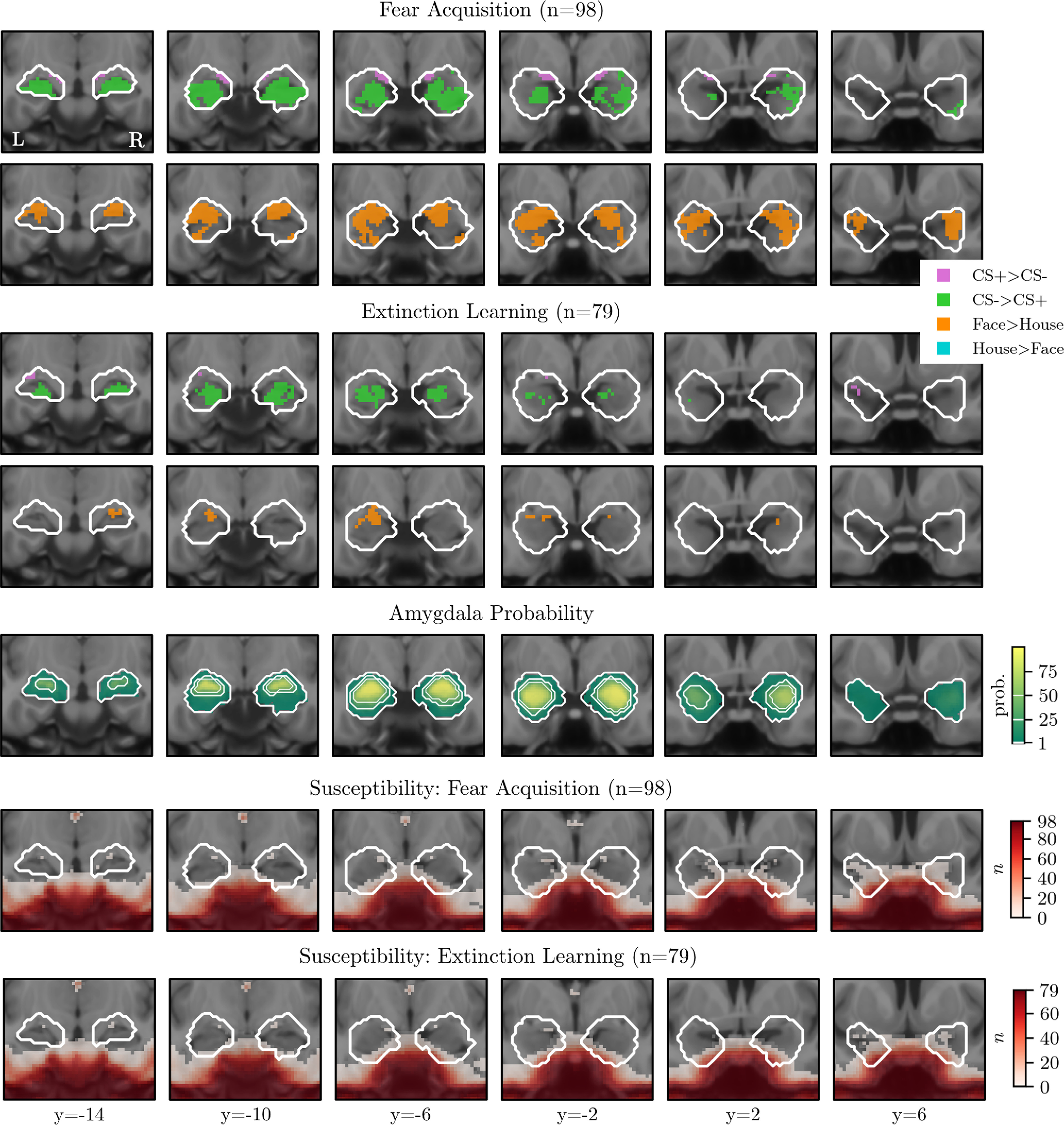
Overview of the voxel-wise ROI analysis. Data were minimally spatially smoothed (2 mm). The top four rows show significant voxels (TFCE corrected, *p* < 0.05) within the amygdala mask (Harvard-Oxford, thresholded at *p* > 0.01), during fear acquisition (rows 1 and 2) and extinction learning (rows 3 and 4). The fifth row depicts the probabilistic ROI, with white outlines representing the different thresholds used to create binary masks. The bottom two rows depict the number of participants who had signal dropout in a particular voxel during fear acquisition and fear extinction. Coordinates refer to MNI space. L, Left; R, right.

Voxel-wise amygdala ROI results (TFCE corrected, *p* < 0.05) for fear acquisition and extinction learning are presented in Figure 2 (2 mm smoothing) and Tables 3 and 4 (2, 5, and 8 mm smoothing). Both during acquisition (Table 3) and extinction learning (Table 4), a small cluster at the border of the superficial nucleus of the amygdala (with a higher probability of being WM or pallidum than amygdala) showed higher activation in response to the CS^+^ compared with the CS^–^ (purple). When thresholding the amygdala masks at *p* > 0.25, no significant voxels remained (Tables 3, 4). In contrast, and as expected, robust responses to faces (averaged over CS^+^ and CS^–^) compared with houses (averaged over CS^+^ and CS^–^) were observed in large parts of the amygdala (up to 75% of the voxels within the mask thresholded at *p* > 0.5; Tables 3, 4).

**Table 3.** Number and percentage of significant voxels during fear acquisition (*n* = 98, TFCE-corrected *p* < 0.05) within each mask, and MNI coordinates of local maxima

	L amygdala (*p* > 0.01) (1452 voxels)	R amygdala (*p* > 0.01) (1623 voxels)	L amygdala (*p* > 0.25) (432 voxels)	R amygdala (*p* > 0.25) (492 voxels)	L amygdala (*p* > 0.50) (240 voxels)	R amygdala (*p* > 0.50) (280 voxels)
CS^+^ > CS^–^						
2 mm	59 (4.1%)	41 (2.5%)	0	0	0	0
	[−16, −2, −10]^*a*^	[14, −14, −10]^*b*^				
5 mm	59 (4.1%)	44 (2.7%)	0	0	0	0
	[−14, −4, −10]^*c*^	[14, −14, −10]^*b*^				
8 mm	50 (3.4%)	40 (2.5%)	0	0	0	0
	[−14, −4, −10]^*c*^	[14, −14, −10]^*b*^				
CS^–^ > CS^+^						
2 mm	395 (27.2%)	566 (34.9%)	187 (43.4%)	249 (50.6%)	126 (52.5%)	161 (57.5%)
	[−20, −14, −18]^*d*^	[28, −16, −16]^*e*^	[−18, −10, −18]^*f*^	[16, −8, −20]^*g*^	[−22, −10, −16]^*h*^	[18, −8, −18]^*i*^
5 mm	399 (27.2%)	585 (36.0%)	181 (41.9%)	248 (50.4%)	127 (52.9%)	171 (61.1%)
	[−20, −14, −20]^*j*^	[32, −16, −18]^*k*^	[−16, −8, −20]^*l*^	[30, −4, −24]^*m*^	[−18, −8, −18]^*n*^	[30, −4, −24]^*m*^
8 mm	434 (29.9%)	646 (39.8%)	192 (44.4%)	278 (56.5%)	127 (52.9%)	195 (69.6%)
	[−20, −16, −18]^*o*^	[32, −16, −18]^*k*^	[−18, −8, −20]^*p*^	[30, −4, −24]^*m*^	[−22, −10, −16]^*h*^	[30, −4, −24]^*m*^
Face > house						
2 mm	618 (42.6%)	564 (34.8%)	309 (71.5%)	273 (55.5%)	191 (79.6%)	180 (64.3%)
	[−18, −6, −14]^*q*^	[20, −6, −14]^*r*^	[−18, −6, −14]^*q*^	[20, −6, −14]^*r*^	[−18, −6, −14]^*q*^	[20, −6, −14]^*r*^
5 mm	645 (44.4%)	651 (40.1%)	309 (71.5%)	295 (60.0%)	197 (82.1%)	194 (69.3%)
	[−18, −6, −14]^*q*^	[20, −6, −14]^*r*^	[−18, −6, −14]^*q*^	[20, −6, −14]^*r*^	[−18, −6, −14]^*q*^	[20, −6, −14]^*r*^
8 mm	710 (48.9%)	710 (43.7%)	340 (78.8%)	321 (65.2%)	208 (86.7%)	211 (75.4%)
	[−20, −6, −14]^*s*^	[20, −6, −14]^*r*^	[−20, −6, −14]^*s*^	[20, −6, −14]^*r*^	[−20, −6, −14]^*s*^	[20, −6, −14]^*r*^
House > face						
2 mm	0	0	0	0	0	0
5 mm	0	0	0	0	0	0
8 mm	0	0	0	0	0	0

Labels associated with local maxima are based on the Harvard-Oxford probabilistic atlases. Some of the peak coordinates fall just outside the amygdala; because of resampling, the anatomical mask at the lowest threshold (*p* > 0.01) is slightly larger than the atlas indicates. L, Left; R, right.

*^a^*53% L cerebral white matter, 27% L cerebral cortex, 10% L pallidum, 5% L amygdala.

*^b^*21% R cerebral white matter.

*^c^*66% L cerebral white matter, 13% L pallidum, 9% L cerebral cortex, 2% L amygdala.

*^d^*92% L hippocampus, 5% L amygdala.

*^e^*93% R hippocampus, 1% R amygdala.

*^f^*58% L hippocampus, 39% L amygdala.

*^g^*63% R hippocampus, 28% R amygdala, 8% R cerebral cortex.

*^h^*64% L amygdala, 21% L hippocampus.

*^i^*58% R amygdala, 40% R hippocampus.

*^j^*99% L hippocampus, 1% L amygdala.

*^k^*96% R hippocampus, 3% R cerebral white matter.

*^l^*58% L hippocampus, 35% L amygdala, 6% L cerebral cortex.

*^m^*60% R amygdala, 27% R hippocampus, 5% R cerebral white matter.

*^n^*69% L amygdala, 30% L hippocampus.

*^o^*95% L hippocampus, 2% L amygdala, 1% L cerebral cortex.

*^p^*55% L hippocampus, 44% L amygdala.

*^q^*90% L amygdala, 3% L cerebral white matter, 2% L cerebral cortex.

*^r^*97% R amygdala, 2% R hippocampus, 1% R cerebral cortex, 1% R cerebral white matter.

*^s^*97% L amygdala, 1% L cerebral cortex, 1% L cerebral white matter.

**Table 4.** Number and percentage of significant voxels during fear extinction learning (*n* = 79, TFCE-corrected *p* < 0.05) within each mask, and MNI coordinates of local maxima

	L amygdala (*p* > 0.01) (1452 voxels)	R amygdala (*p* > 0.01) (1623 voxels)	L amygdala (*p* > 0.25) (432 voxels)	R amygdala (*p* > 0.25) (492 voxels)	L amygdala (*p* > 0.50) (240 voxels)	R amygdala (*p* > 0.50) (280 voxels)
CS^+^ > CS^–^						
2 mm	15 (1.0%)	0	0	0	0	0
	[−28, −14, −10]^*a*^					
	5 (0.3%)					
	[−28, 6, −18]^*b*^					
	1 (0.1%)					
	[−18, −2, −10]^*c*^					
5 mm	22 (1.5%)	0	0	0	0	0
	[−30, 6, −14]^*d*^					
	14 (1.0%)					
	[−30, −16, −10]^*e*^					
8 mm	29 (2.0%)	0	0	0	0	0
	[−32, 6, −16]^*f*^					
	4 (0.3%)					
	[−30, −16, −10]^*e*^					
CS^–^ > CS^+^						
2 mm	193 (13.3%)	157 (9.7%)	137 (31.7%)	77 (15.7%)	106 (44.2%)	55 (19.6%)
	[−18, −12, −22]^*g*^	[22, −10, −16]^*h*^	[−18, −8, −20]^*i*^	[22, −10, −16]^*h*^	[−16, −6, −20]^*j*^	[22, −6, −20]^*k*^
5 mm	215 (14.8%)	147 (9.1%)	136 (31.5%)	67 (13.6%)	105 (43.8%)	50 (17.9%)
	[−18, −12, −22]^*g*^	[22, −8, −16]^*l*^	[−16, −8, −20]^*m*^	[22, −8, −16]^*l*^	[−28, −4, −20]^*n*^	[22, −8, −16]^*l*^
8 mm	197 (13.6%)	132 (8.1%)	140 (32.4%)	61 (12.4%)	100 (41.7%)	42 (15.0%)
	[−18, −12, −22]^*g*^	[20, −8, −20]^*o*^	[−20, −6, −22]^*p*^	[20, −8, −20]^*o*^	[−20, −6, −22]^*p*^	[20, −6, −20]^*k*^
Face > house						
2 mm	81 (5.6%)	21 (1.3%)	114 (26.4%)	28 (5.7%)	103 (42.9%)	23 (8.2%)
	[−18, −8, −14]^*q*^	[28, −16, −12]^*r*^	[−18, −8, −14]^*q*^	[24, 2, −18]^*s*^	[−18, −8, −14]^*q*^	[24, 2, −18]^*s*^
		3 (0.1%)		3 (0.6%)		
		[24, 2, −18]^*s*^		[26, −14, −12]^*v*^		
		1 (0.1%)				
		[20, −2, −14]^*t*^				
		1 (0.1%)				
		[12, −4, −10]^*u*^				
5 mm	69 (4.8%)	96 (5.9%)	112 (25.9%)	91 (18.5%)	105 (43.8%)	67 (23.9%)
	[−20, −8, −14]^*w*^	[30, −18, −10]^*x*^	[−20, −8, −14]^*w*^	[24, 2, −18]^*s*^	[−20, −8, −14]^*w*^	[24, 2, −18]^*s*^
8 mm	70 (4.8%)	70 (4.3%)	128 (29.6%)	100 (20.3%)	126 (52.5%)	77 (27.5%)
	[−20, −8, −14]^*w*^	[20, −2, −10]^*y*^	[−20, −8, −14]^*w*^	[20, −2, −12]^*bb*^	[−20, −8, −14]^*w*^	[20, −2, −12]^*bb*^
		41 (2.5%)				
		[30, −16, −12]^*z*^				
		1 (0.1%)				
		[24, −8, −16]*^aa^*				
House > face						
2 mm	0	0	0	0	0	0
5 mm	0	0	0	0	0	0
8 mm	0	0	0	0	0	0

Labels associated with local maxima are based on the Harvard-Oxford probabilistic atlas. Some of the peak coordinates fall just outside the amygdala, because - because of resampling - the anatomical mask at the lowest threshold (*p* > 0.01) is slightly larger than the atlas indicates. R, Right; L, left.

*^a^*71% L cerebral white matter, 17% L putamen, 6% L amygdala, 3% L hippocampus, 1% L pallidum.

*^b^*78% L cerebral cortex, 2% L amygdala.

*^c^*51% L cerebral white matter, 32% L cerebral cortex, 7% L amygdala, 7% L pallidum.

*^d^*77% L cerebral cortex, 20% L cerebral white matter.

*^e^*74% L cerebral white matter, 19% L putamen, 4% L hippocampus, 1% L amygdala.

*^f^*82% L cerebral cortex, 8% L cerebral white matter, 1% L amygdala.

*^g^*87% L hippocampus, 9% L cerebral cortex, 3% L cerebral white matter, 1% L amygdala.

*^h^*47% R amygdala, 34% R hippocampus.

*^i^*55% L hippocampus, 44% L amygdala.

*^j^*63% L amygdala, 33% L hippocampus, 3% L cerebral cortex.

*^k^*74% R amygdala, 25% R hippocampus.

*^l^*77% R amygdala, 14% R hippocampus.

*^m^*58%L hippocampus, 35% L amygdala, 6% L cerebral cortex.

*^n^*96% L amygdala, 3% L hippocampus, 1% L cerebral white matter, 1% L cerebral cortex.

*^o^*64% R hippocampus, 34% R amygdala.

*^p^*60% L amygdala, 40% L hippocampus.

*^q^*92% L amygdala, 4% L cerebral white matter, 1% L hippocampus.

*^r^*40% R hippocampus, 21% R cerebral white matter, 11% R amygdala,.

*^s^*67% R amygdala, 21% R cerebral cortex, 0% Right cerebral white matter.

*^t^*72% R amygdala, 17% R cerebral cortex, 2% Right cerebral white matter.

*^u^*64% R cerebral white matter, 9% Right pallidum, 3% R cerebral cortex.

*^v^*31% R amygdala, 20% R cerebral white matter, 17% R hippocampus.

*^w^*96% L amygdala, 1% L cerebral white matter, 1% L hippocampus.

*^x^*65% R cerebral white matter, 24% R hippocampus, 2% R amygdala.

*^y^*45% R cerebral white matter, 25% R cerebral cortex, 25% R amygdala, 4% R pallidum.

*^z^*41% R cerebral white matter, 39% R hippocampus, 6% R amygdala.

*^aa^*76% R amygdala, 12% R hippocampus.

*^bb^*50% R amygdala, 30% R cerebral cortex, 13% R cerebral white matter, 2% R pallidum.

Unexpectedly, the amygdala showed much stronger responses to the CS^–^ compared with the CS^+^ (Tables 3, 4). Further inspection suggested that some of the signal originated from the anterior hippocampus/amygdala–hippocampus transition area, which is in line with other studies (Fullana et al., 2016), though a substantial part of the signal seemed to originate from the basolateral part of the amygdala.

To examine signal dropout more directly, Figure 2 also depicts the number of participants who had signal dropout in a particular voxel during fear acquisition and fear extinction. All participants showed adequate signal in the amygdala mask thresholded at *p* > 0.5. Some participants had dropout in regions with a low probability of being part of the amygdala nuclei. To estimate the impact of signal dropout, we repeated the voxel-wise ROI analysis while excluding any participants with >5% dropout in left and/or right amygdala (*n* = 12 for acquisition; *n* = 7 for extinction). This did not change the pattern of results (parametric maps available on OSF; https://osf.io/cq5zr/). In sum, from the voxel-wise ROI analyses it is evident that a reliable signal was measured in the amygdala, and that the absence of robust responses to learned threat cannot be attributed to a poor signal.

**Figure 3.**
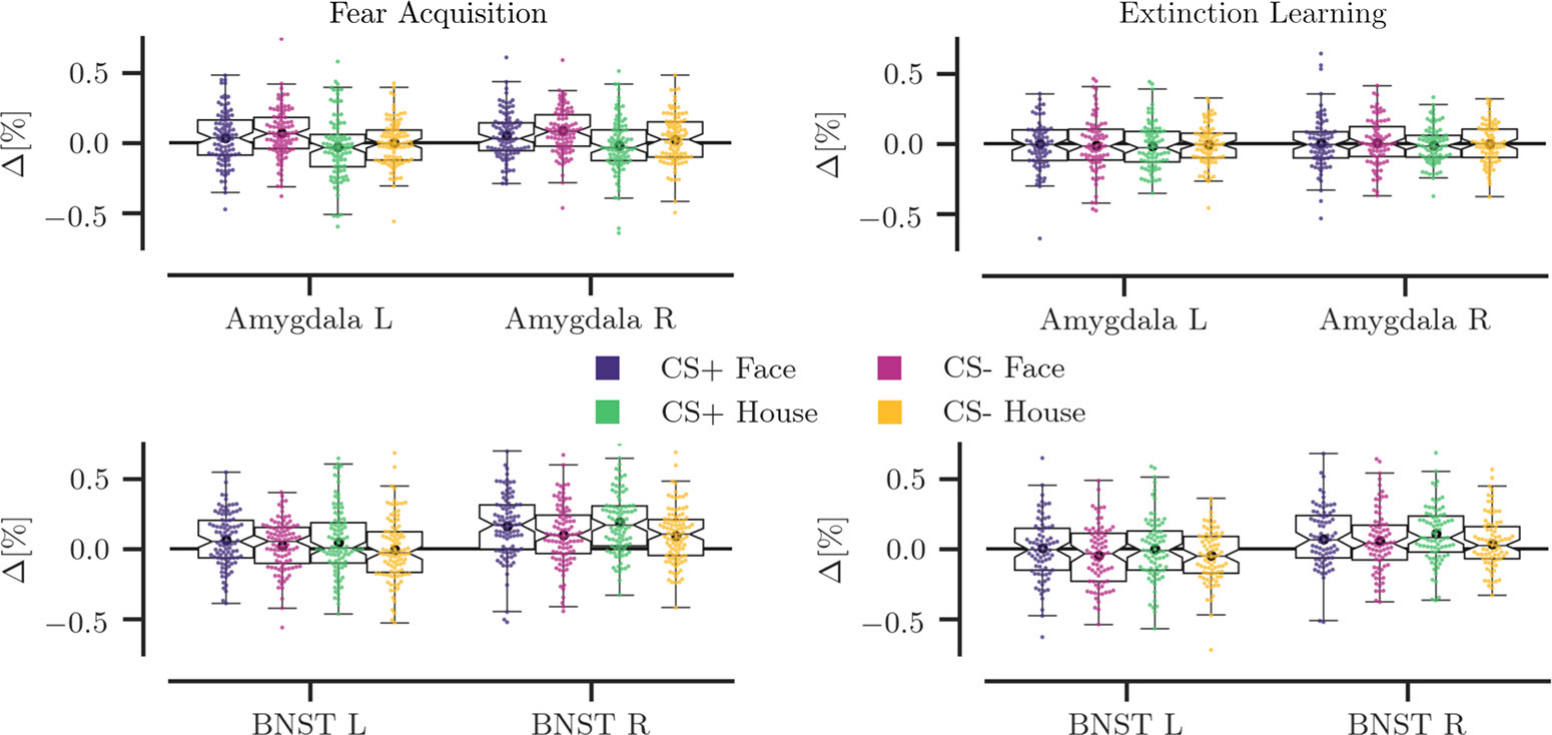
Overview of the ROI analysis of the mean signal change during fear acquisition and extinction learning. The top graph depicts the signal change per individual in the left and right amygdala per condition, and the bottom graph shows signal change per individual in the left and right BNST. During fear acquisition, amygdala responses are stronger to faces than to houses, and stronger to the CS^–^ than the CS^+^, while no significant differences were observed during extinction learning. In contrast, the BNST shows typical threat anticipation with higher responses to the CS^+^ than the CS^–^ during both experimental phases. L, Left; R, right.

In addition to these voxel-wise analyses, we averaged activity at the ROI level (mask thresholded at *p* > 0.01) per condition, per individual, to directly compare the difference between face and house and CS^+^ and CS^–^ (Fig. 3). During fear acquisition, a main effect of picture type confirmed that faces elicited significantly more activation than houses in the left amygdala (*F*_(1,97)_ = 21.08, *p* < 0.001, η_P_^2^ = 0.18) and right amygdala (*F*_(1,97)_ = 21.16, *p* < 0.001, η_P_^2^ = 0.18). In the left amygdala, the difference between CS^+^ and CS^–^ was not significant (*F*_(1,97)_ = 2.79, *p* = 0.098, η_P_^2^ = 0.03), while in the right amygdala the CS^–^ evoked more activation than the CS^+^ (*F*_(1,97)_ = 6.82, *p* = 0.010, η_P_^2^ = 0.07). There was also no significant interaction between learned threat and picture type (*p* values > 0.759). A direct comparison of the positive differences between face and house stimuli in left (mean = 0.069, SD = 0.148) and right (mean = 0.065, SD = 0.140) amygdala with the negative differences between CS^+^ and CS^–^ in left (mean = −0.031, SD = 0.182) and right (mean = −0.041, SD = 0.155) amygdala revealed a significant difference (*t*_(97)_ = 3.57, *p* = 0.001; and *t*_(97)_ = 4.35, *p* < 0.001, respectively). Notably, during extinction learning, there were no main effects of picture type, main effects of threat, or interactions between picture type and threat (all *p* values > 0.386). This tentatively suggests that the amygdala mainly favors social information (faces), and potentially safety information (CS^–^), when it is novel (Blackford et al., 2010, 2013).

Interestingly, results in the BNST, which is part of the “extended amygdala” circuitry (Shackman and Fox, 2016), did show the expected conditioning effects, with stronger responses to CS^+^ compared with CS^–^ stimuli (Fig. 3), in left (*F*_(1,97)_ = 6.23, *p* = 0.014, η_P_^2^ = 0.06) and right (*F*_(1,97)_ = 18.61, *p* < 0.001, η_P_^2^ = 0.16) BNST. This is in line with other large-scale studies of fear conditioning (Klumpers et al., 2017) and threat anticipation (Hur et al., 2020). During extinction, the difference between CS^+^ compared with CS^–^ stimuli was still significant in the left BNST (*F*_(1,78)_ = 4.45, *p* = 0.038, η_P_^2^ = 0.05), and marginally significant in the right BNST (*F*_(1,78)_ = 3.76, *p* = 0.056, η_P_^2^ = 0.05).

### Whole-brain results

**Figure 4.**
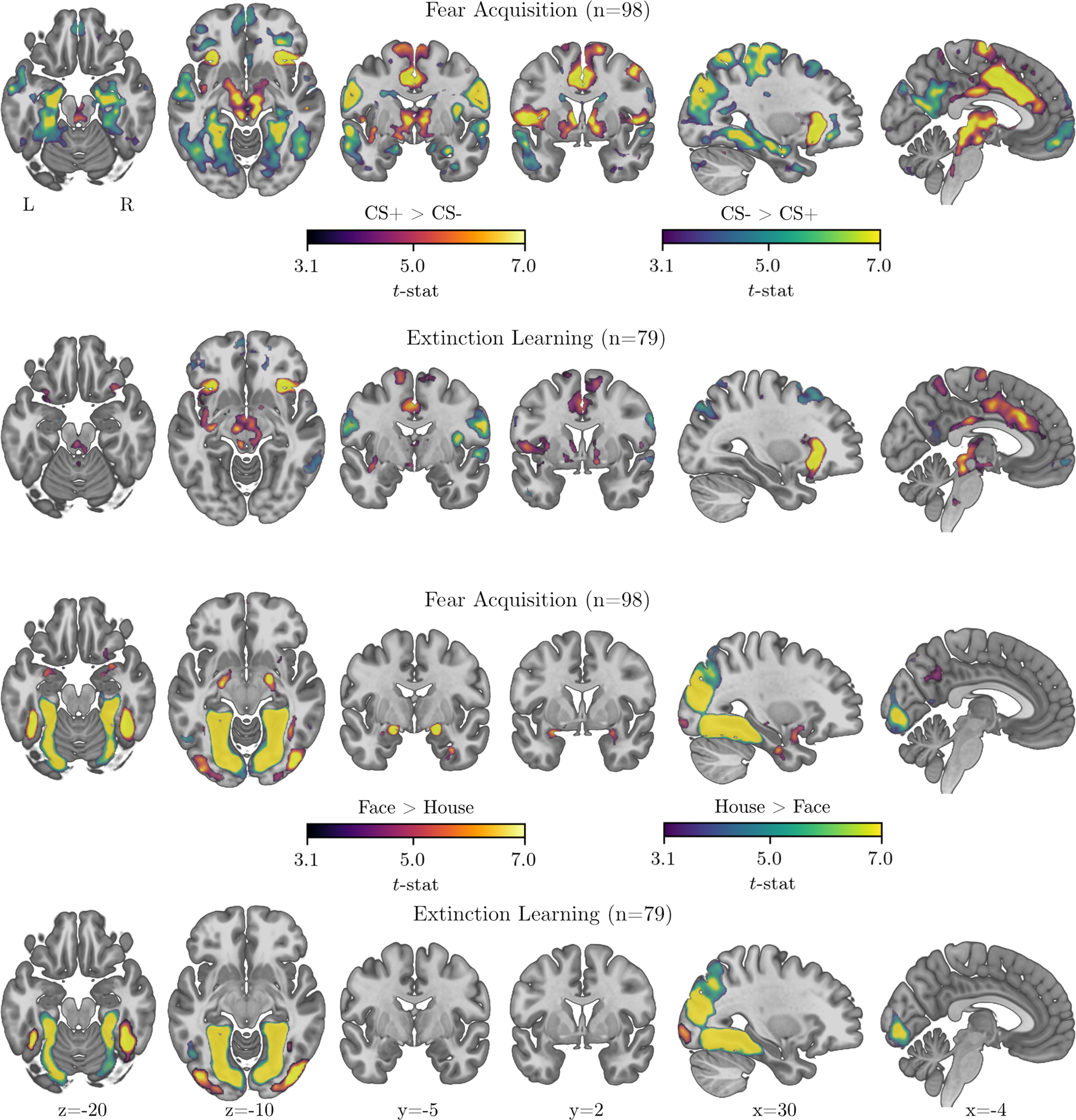
Whole-brain results for the CS^+^ versus CS^–^ contrast (top rows), and the face versus house contrast (bottom rows), during fear acquisition and extinction learning (TFCE corrected, *p* < 0.05). Results for the CS^+^ > CS^–^ comparison and for the face > house comparison appear in purple/orange/yellow colors. Results for the CS^–^ > CS^+^ comparison and for the house > face comparison are shown in blue/green/yellow colors.

To allow comparison with previous meta-analyses, whole-brain results for the contrast CS^+^ versus CS^–^ are displayed in Figure 4 and summarized in Table 5. Compared with the CS^–^, the CS^+^ elicited more activation in areas corresponding to the salience network, including the thalamus, brainstem, striatum, temporoparietal junction, anterior insula (extending to frontal opercular and frontal orbital cortex), and a large cluster centered around the midcingulate cortex (following nomenclature by Vogt and Paxinos, 2014; van Heukelum et al., 2020; in the human fear-learning literature, it is most commonly referred to as “dorsal anterior cingulate”), extending to the anterior cingulate cortex and the superior frontal gyrus, both during fear acquisition and fear extinction learning. These results are in line with previous meta-analyses on fear acquisition (Mechias et al., 2010; Fullana et al., 2016) and extinction learning (Fullana et al., 2018). The reverse contrast primarily showed activation in the hippocampus extending into the posterior parts of the amygdala, and occipital and temporal areas, and a cluster at the intersection of frontal pole and medial prefrontal cortex.

**Table 5.** Brain areas showing differential activation for the contrast CS^+^ versus CS^–^

Brain region (COG)	MNI coordinates			Volume (voxels)	Maximum *z*
	*x*	*y*	*z*		
CS^+^ > CS^–^					
Acquisition (*n* = 98)					
L and R cingulate gyrus, anterior division (midcingulate cortex)/paracingulate cortex/superior frontal gyrus/cingulate gyrus, posterior division/juxtapositional lobule	−4	22	24	6904	9.3
L and R caudate/thalamus/brainstem	−8	8	4	4313	8.9
L frontal orbital cortex/anterior insular cortex/frontal operculum cortex/precentral gyrus/inferior frontal gyrus	−32	26	−8	2570	11.5
R frontal orbital cortex/anterior insular cortex/frontal operculum cortex/precentral gyrus/inferior frontal gyrus	32	24	−8	2524	12.2
L parietal operculum cortex/supramarginal gyrus	−56	−30	22	844	8.81
R supramarginal gyrus/angular gyrus	62	−44	24	464	8.24
L superior parietal lobule/postcentral gyrus	−20	−50	72	434	6.55
R inferior frontal gyrus/middle frontal gyrus	38	14	26	262	4.71
R precentral gyrus/middle frontal gyrus	46	2	50	257	6.88
R parietal operculum cortex /supramarginal gyrus, anterior division	54	−26	26	210	7.46
R cerebellum	34	−54	−28	46	5.79
R middle temporal gyrus	56	−24	−8	23	6.68
Extinction (*n* = 79)					
L and R cingulate gyrus, anterior division (midcingulate cortex)/paracingulate cortex/superior frontal gyrus/cingulate gyrus, posterior division/juxtapositional lobule	−4	24	32	4168	7.18
L frontal orbital cortex/anterior insular cortex/frontal operculum cortex/precentral gyrus	−32	24	−6	2266	8.64
R frontal orbital cortex/anterior insular cortex/frontal operculum cortex/precentral gyrus/inferior frontal gyrus	34	24	−6	2136	10.6
L and R brainstem/thalamus	−4	−30	−14	1667	6.59
L superior parietal lobule	−20	−50	72	962	7.25
L parietal operculum cortex/supramarginal gyrus	−58	−34	26	530	8.44
L superior frontal gyrus/juxtapositional lobule	−12	−10	72	490	5.71
R parietal operculum cortex/supramarginal gyrus	54	−26	26	398	5.92
L caudate	−6	8	2	207	6.08
Brainstem	0	−38	−34	108	5.3
R cerebellum	42	−54	−30	54	4.49
R lingual gyrus	2	−64	−4	44	5.41
R precuneus cortex	8	−52	64	13	3.53
CS^–^ > CS^+^					
Acquisition (*n* = 98)					
L and R postcentral gyrus/precentral gyrus/superior parietal lobule/supramarginal gyrus/central opercular cortex/insular cortex/planum temporale/lingual gyrus/lateral occipital cortex/inferior temporal gyrus/temporal, occipital fusiform gyrus/parahippocampal gyrus/hippocampus/amygdala	−58	−14	38	39,682	10.8
L superior frontal gyrus/middle frontal gyrus/frontal pole	−22	26	50	3681	7.21
R frontal pole	30	38	−10	1012	6.44
R superior frontal gyrus/middle frontal gyrus/frontal pole	26	26	54	728	5.75
R cerebellum	22	−86	−38	323	5.21
L frontal orbital cortex/frontal pole	−40	30	−14	312	6.23
R temporal fusiform cortex/temporal pole	38	−8	−38	238	5.53
R frontal pole	38	38	18	140	4.61
L inferior frontal gyrus/white matter	−28	24	18	122	4.47
L frontal pole	−46	44	6	81	4.07
L and R subcallosal cortex	2	22	−10	76	4.65
L cerebellum	−8	−72	−46	74	3.99
L white matter	−10	26	4	43	3.76
L frontal pole	−22	50	−6	33	4.64
R temporal pole	34	22	−32	32	4.09
R white matter	34	−38	24	26	3.85
L precentral gyrus	−8	−22	50	23	4.31
L superior frontal gyrus	−20	−12	50	19	3.93
White matter	0	4	20	18	4.08
R cerebellum	20	−62	−44	16	4.01
L parahippocampal gyrus	−20	0	−40	15	4.53
L white matter	−18	36	−2	14	3.61
L middle frontal gyrus	−44	12	38	12	3.89
L superior frontal gyrus	−22	−6	60	11	3.62
R frontal pole	16	42	36	11	3.53
Extinction (*n* = 79)					
R postcentral gyrus/precentral gyrus/superior parietal lobule/supramarginal gyrus	60	−6	24	2377	6.72
L postcentral gyrus/precentral gyrus/superior parietal lobule/supramarginal gyrus/central opercular cortex	−54	−8	26	1561	6.11
R lateral occipital cortex, superior division	50	−70	30	865	4.95
L middle frontal gyrus/superior frontal gyrus	−32	16	60	813	5.19
R middle frontal gyrus/superior frontal gyrus	30	24	52	684	4.89
L lateral occipital cortex, superior division	−30	−74	30	655	4.36
L middle frontal gyrus/inferior frontal gyrus	−52	28	26	643	5.1
L frontal pole	−8	60	−8	345	4.73
R lateral occipital cortex, superior division	20	−70	64	322	5.41
R inferior temporal gyrus/middle temporal gyrus	52	−52	−8	302	4.6
R central opercular cortex/insula cortex	42	−8	16	245	8.55
L precuneus cortex	−8	−54	16	195	5
L frontal pole	−8	66	20	78	4.89
L temporal pole	−48	2	−38	40	4.73
R temporal pole	46	18	−36	37	4.17
L hippocampus	−18	−12	−22	35	5.44
R precuneus cortex	10	−54	16	30	4
R postcentral gyrus	8	−34	72	29	4.87
R precentral gyrus	6	−28	56	24	4.69
L lateral occipital cortex, superior division	−22	−64	58	23	3.6
R postcentral gyrus	44	−38	66	21	3.93
R middle temporal gyrus, posterior division	70	−14	−16	19	4.18
R precentral gyrus	26	−26	50	16	3.57
L lateral occipital cortex, superior division	−26	−86	20	15	3.46
R middle temporal gyrus, anterior division	52	0	−32	15	3.98
L temporal pole	−48	14	−30	12	5.06

Whole-brain activation (TFCE corrected, *p* < 0.05) that discriminates the threat-associated (CS^+^) stimuli from the control stimuli (CS^–^). Coordinates are in MNI space and indicate the voxel with the highest *z* value, for each significant cluster. Minimum cluster size reported here: *k* > 10. Labels are derived from the Harvard-Oxford cortical and subcortical atlases, and Vogt and Paxinos (2014), specifically for the cingulate cortex. L, Left; R, right; COG, Center of Gravity.

Whole-brain results for the face versus house contrast are displayed in Figure 4 and summarized in Table 6. Typical activation is seen in the fusiform face area (located in the temporal occipital fusiform cortex), in response to faces (Kanwisher et al., 1997), and in the parahippocampal place area (located in the posterior division of the parahippocampal gyrus) in response to houses (Epstein and Kanwisher, 1998). Notably, during fear acquisition a large cluster of activation in the amygdala was observed in response to faces compared with houses, in a region overlapping with coordinates previously reported in the context of learned threat (Sjouwerman et al., 2020) and other forms of certain threat anticipation (Hur et al., 2020). During extinction learning, activation in the amygdala did not reach significance (only in voxel-wise ROI analyses, Table 4), suggesting an interaction of socially relevant information and novelty (Blackford et al., 2010, 2013).

**Table 6.** Brain areas showing differential activation for the contrast face versus house

Brain region (COG)	MNI coordinates			Volume (voxels)	Maximum *z*
	*x*	*y*	*z*		
Face > house					
Acquisition (*n* = 98)					
R lateral occipital cortex, inferior division/occipital pole	50	−70	0	2747	11.9
L lateral occipital cortex, inferior division/occipital pole	−42	−90	−12	2283	6.96
R precuneus cortex/posterior cingulate cortex	2	−62	32	830	6.35
R temporal occipital fusiform cortex/temporal fusiform cortex, posterior division	42	−52	−18	714	12.3
R amygdala/insular cortex/frontal orbital cortex	20	−6	−14	462	12.2
L temporal occipital fusiform cortex/temporal fusiform cortex, posterior division	−40	−50	−18	409	9.04
L amygdala	−18	−6	−14	295	9.43
R parahippocampal gyrus, anterior division	32	−10	−32	190	6.94
L and R frontal pole	0	62	−16	56	5.05
L and R subcallosal cortex	4	22	−14	39	5.35
L parahippocampal gyrus, anterior division	−32	−10	−32	14	5.73
R ventricle	24	−44	16	13	5.41
Extinction (*n* = 79)					
R temporal occipital fusiform cortex/temporal fusiform cortex, posterior division/lateral occipital cortex, inferior division/middle temporal gyrus/occipital pole	42	−52	−18	2860	13.1
L lateral occipital cortex, inferior division/occipital pole	−44	−82	−8	456	6.94
L temporal occipital fusiform cortex/temporal fusiform cortex, posterior division	−40	−48	−20	127	7.33
House > face					
Acquisition (*n* = 98)					
L and R temporal occipital fusiform cortex/lingual gyrus/occipital fusiform gyrus/parahippocampal gyrus/occipital pole/lateral occipital cortex, superior division/inferior temporal gyrus	28	−54	−12	17,682	23.4
L lateral occipital cortex, inferior division/inferior temporal gyrus, temporooccipital part/middle temporal gyrus	−46	−62	−6	144	5.16
R inferior temporal gyrus, temporo-occipital part/middle temporal gyrus	58	−50	−14	38	3.91
R cerebellum	40	−40	−34	33	4.34
Extinction (*n* = 79)					
L and R temporal occipital fusiform cortex/lingual gyrus/occipital fusiform gyrus/parahippocampal gyrus/occipital pole/lateral occipital cortex, superior division/inferior temporal gyrus	28	−54	−12	15,463	17.2
L lateral occipital cortex, inferior division/inferior temporal gyrus, temporooccipital part/middle temporal gyrus	−48	−62	−8	237	5.95
L thalamus	−20	−28	0	107	5.82
R cingulate gyrus, posterior division	6	−38	42	69	4.92
L superior parietal lobule/supramarginal gyrus/lateral occipital cortex, superior division	−28	−44	38	30	4.38
R thalamus	22	−30	6	16	5.17

Whole-brain activation (TFCE corrected, *p* < 0.05) that discriminates the face stimuli from house stimuli. Coordinates are in MNI space and indicate the voxel with the highest *z* value for each significant cluster. Minimum cluster size reported here: *k* > 10. Labels are derived from the Harvard-Oxford cortical and subcortical atlases. L, Left; R, right; COG, Center of Gravity.

### Relation between amygdala and indices of fear

Average trait anxiety in our sample, as measured by the State-Trait Anxiety Inventory–Trait version (STAI-T; Table 1), was comparable to average trait anxiety in a recent large-sample imaging study (Sjouwerman et al., 2020). As trait anxiety has been reported to covary with amygdala activation in Pavlovian conditioning (Sjouwerman et al., 2020), we entered demeaned scores on the STAI-T as a separate predictor in our voxel-wise amygdala analysis (probability threshold, *p* > 0.01). No significant voxels were found (parametric map for this control analysis available on OSF; https://osf.io/cq5zr/).

In many fear-conditioning studies, participants are excluded if they do not show evidence of learning as assessed by an independent measure, such as skin conductance or pupil dilation. The use of idiosyncratic criteria to define such “non-learners” has been criticized (Lonsdorf et al., 2019), and poses problems for replication studies. The definition of nonlearners on the basis of a single outcome measure is especially not recommended. Here, we nevertheless intended to examine the effects for learners and nonlearners separately, given that the purpose of our article was to better understand conflicting findings with regard to amygdala activation. However, there were only three participants who did not show higher pupil dilation in response to the CS^+^ compared with the CS^–^, averaged across all acquisition trials. Instead, we added differential pupil dilation per individual as a separate predictor in a voxel-wise amygdala analysis (probability threshold, *p* > 0.01), to test whether stronger indices of anticipatory arousal (pupil dilation, CS^+^ > CS^–^ all acquisition or extinction trials) were associated with stronger amygdala activation, during fear and extinction learning. This was not the case (parametric map for this control analysis available on OSF; https://osf.io/cq5zr/).

## Discussion

In this study, we tested the hypothesis that fear and extinction learning in humans leads to BOLD activation in the amygdala, provided there is a good signal in this area. Analyzing fear-conditioning data from 98 participants, we found little evidence for activation in response to CS^+^ compared with CS^–^ stimuli in the amygdala, despite robust physiological evidence of fear and extinction learning (differential pupil dilation). In fact, large parts of the amygdala responded more strongly to the CS^–^ compared with the CS^+^ during both fear acquisition and extinction learning, suggesting involvement in safety processing or inhibition of fear. In contrast, many other brain areas, such as the midcingulate cortex, anterior insula, and BNST (part of the extended amygdala; Shackman and Fox, 2016), did show activation in response to learned threat. Crucially, there was negligible signal dropout, and, using the same data, we showed robust amygdala activation in response to face compared with house stimuli, indicating that our fMRI sequence managed to obtain a reliable signal in this area.

Both the absence of effects in the amygdala and the presence of effects in the midcingulate cortex and anterior insula are in line with the literature on human fear and extinction learning (Sehlmeyer et al., 2009; Mechias et al., 2010; Fullana et al., 2016, 2018). Still, some studies do report amygdala activation. With regard to older studies, there seems to be a publication bias, for example, a tendency to report uncorrected and weak effects in the amygdala while ignoring strong effects in other brain areas, or including clusters that have a higher probability of belonging to white matter or neighboring regions such as the putamen, pallidum, and hippocampus. However, this does not explain why recent studies leveraging large samples and state-of-the-art methods report amygdala activation in Pavlovian fear learning (e.g., Sjouwerman et al., 2020). Over the years, numerous explanations for conflicting findings have been proposed (Sehlmeyer et al., 2009; Mechias et al., 2010; Fullana et al., 2016, 2018, 2020; Shackman and Fox, 2021), summarized as (1) insufficiently “fear”-provoking conditioning procedures; (2) heterogeneity of experimental designs and analytical methods; and (3) the functional heterogeneity of the amygdala yielding small effects, combined with small study samples (forming the basis for meta-analyses, which also typically exclude results from ROI approaches), leading to lack of statistical power. Below, we will discuss how each of these points relate to the present study.

First, for ethical reasons, human fear-conditioning procedures can only use USs that are moderately aversive, and relatively controllable. While the present study is comparable to other studies regarding shock intensity, and successful fear conditioning and extinction were evident from both pupil dilation and BOLD responses outside the amygdala, the fear elicited by the CS is likely quite different from the distress experienced by nonhuman animals (LeDoux, 2014; Fullana et al., 2016; Haaker et al., 2019).

Second, design parameters and stimulus material may influence amygdala activation in Pavlovian conditioning in humans (and substantially differ from those used in animals; Haaker et al., 2019). Thus far, no pattern has been identified (or systematically investigated) regarding the impact of CS modality (visual, acoustic, olfactory), fear relevance of the CS, threat imminence, uncertainty/certainty of threat (full vs partial reinforcement and, relatedly, US-related confounds), or the inclusion of concurrent measurements (e.g., startle potentiation, online expectancies), though the number of trials and the modality of the US did not appear to have a large impact (Fullana et al., 2016). Furthermore, task-related changes in heart rate may induce spurious activation, given the proximity of the amygdala to large veins (Boubela et al., 2015). Another source of variance stems from task instructions, ranging from participants being told that they might receive shocks during the experiment (Reddan et al., 2018; Sjouwerman et al., 2020), to more explicit instructions about the differential contingencies (the present study; Hermans et al., 2016), to instructed fear learning, where participants are told beforehand which stimulus is followed by a shock and which is not (Phelps et al., 2001; Klumpers et al., 2017; sample 2). Recent large-sample studies on anxiety (without an associative learning component) showed that both certain and uncertain threat anticipation (compared with safety) elicit activation in the dorsal amygdala (Hur et al., 2020, 2021), with certain threats eliciting more activation than uncertain threats. Amygdala deactivation to uncertain threat has also been observed, either in rostral parts only (Hur et al., 2020), or in multiple amygdala nuclei (Morrow et al., 2021). This illustrates the functional heterogeneity of the amygdala, as well as the potential impact of shock predictability, and thus, indirectly, of task instructions. Furthermore, task instructions influence learning rates (i.e., less explicit instructions increase the likelihood that participants remain unaware of the contingencies, and/or fail to show differential physiological responses to threat). In turn, this might affect (the need for) exclusion of subjects (nonlearners) from analyses and, consequently, outcomes (Lonsdorf et al., 2019). Although an early meta-analysis of fear-conditioning studies (Mechias et al., 2010) reported a lack of robust amygdala activation regardless of protocol (instructed or uninstructed fear), a meta-analysis of more recent, large-sample studies is needed to systematically re-evaluate the effect of task instructions. Finally, studies differ in which trials are used to examine threat-related responses, with some averaging across all trials (as we do) and others analyzing early and late phases separately. This is relevant given the mechanistically informed hypothesis and some evidence that the amygdala is only active in the initial stages of fear learning (Büchel et al., 1998; LaBar et al., 1998; Lindner et al., 2015). Although this was not confirmed in a meta-analysis comparing early versus late acquisition (Fullana et al., 2016), results may differ depending on how “early” is defined (Lonsdorf et al., 2019). Alternatively, it may be that the amygdala is not so important for predicting threat as it is for evaluating the outcome of a prediction. Analysis of a large sample (*n* = 173) showed that while amygdala activation was not observed during shock anticipation (CS^+^ > CS^–^), strong responses to shock delivery (i.e., the US) were observed (after rigorous correction for confounds; Klumpers et al., 2017). Although such responses could merely reflect pain, they may also reflect the preference of the amygdala for opportunities for learning, as other research suggests that neuronal plasticity (McNally et al., 2011) and BOLD activation (Michely et al., 2020) in the basolateral amygdala are strongest when the magnitude or occurrence of the US is unexpected.

A third explanation for lack of amygdala activation in human fear learning posits that the relatively low spatial resolution of fMRI (e.g., compared with local field potentials) may be insufficient to study structures like the amygdala, which includes nuclei with dissociable and even opposite function (Swanson and Petrovich, 1998; Reijmers et al., 2007; Quirk and Mueller, 2008; Ciocchi et al., 2010; Haubensak et al., 2010; Orsini and Maren, 2012). In this context, it is important to distinguish Pavlovian conditioning from other types of salience processing. Robust amygdala activation has been observed across a range of different tasks including anticipation or occurrence of unpredictable/predictable aversive stimuli (Hur et al., 2020; Michely et al., 2020; Sambuco et al., 2020), pleasant stimuli such as erotica and reward (Lindquist et al., 2012, 2016), and socially relevant stimuli (Bickart et al., 2014). Detection of responses at the voxel level requires a relatively uniform response from the underlying neuronal code (e.g., ∼50% of amygdala neurons respond to faces; Rutishauser et al., 2015), which is hard to obtain with the sparsely distributed neurons underlying fear memory (Reijmers et al., 2007). Within each voxel, different signals may cancel each other out, and even if they do not, a single activated voxel may disappear with smoothing (which we only did lightly), or with multiple-comparisons correction.

Alternative approaches may offer a solution to some of the challenges mentioned above. For example, while ultra high-resolution BOLD-MRI does not approach the level of neurons, it does seem to offer a somewhat finer-grained mapping of microcircuits involving amygdala subnuclei (Saygin et al., 2017; Torrisi et al., 2018). In addition, analytical approaches such as multivoxel pattern analysis (MVPA) may provide higher sensitivity compared with univariate analyses of BOLD activation in detecting changes related to fear learning and memory, as shown in numerous regions including the amygdala (Bach et al., 2011; Visser et al., 2013, 2015; Braem et al., 2017). MVPA assesses distributed BOLD patterns to characterize the distinctive neural representation of a stimulus or condition. These patterns are used either for (binary) classification analysis or (continuous) representational similarity analysis (RSA; Kriegeskorte et al., 2008). Crucially, patterns are not restricted to voxels that reach a statistical threshold: subthreshold activation and nonresponding voxels can be equally informative, enabling the detection of sparse memory traces (Bach et al., 2011). Relatedly, MVPA does not require, or imply, that voxels preferentially respond to the CS^+^. In fact, previously observed differential pattern similarity in the amygdala (Visser et al., 2013, 2015) was likely driven by higher responses to the CS^–^. While dissociable amygdala patterns suggest involvement in fear learning and extinction, the fact that they are driven by stronger responses to learned safety does not fit with how the amygdala is typically portrayed in the literature (but see Genud-Gabai et al., 2013; Morrow et al., 2021).

Aside from being more sensitive, MVPA and RSA can be used to ask a different kind of question: that is, how something is represented in the brain, rather than where exactly the signal originates (Haxby, 2012). This has been leveraged to study the formation, enhancement, persistence, generalization, and extinction of fear memory (Li et al., 2008; Bach et al., 2011; Visser et al., 2011, 2013, 2015, 2016; Hauner et al., 2013; Dunsmoor et al., 2014; de Voogd et al., 2016; Braem et al., 2017; Koizumi et al., 2017; Gerlicher et al., 2018; Reddan et al., 2018; Undeger et al., 2020). Importantly, RSA is relatively independent of methodology (e.g., input can also be electrophysiology; conceptual models), making it a powerful tool for identifying higher-order isomorphisms in representational geometries (e.g., between humans and other primates; Kriegeskorte, 2008) and factors influencing these geometries across species. This opens up avenues for addressing many exciting translational questions on the acquisition and extinction of fear.

### Conclusion

While the amygdala is generally regarded as the integrative center of the brain for fear learning, at present this is not strongly corroborated by neuroimaging evidence in humans. Whether this is because the field needs better methods and more data, because neural processes take place at a different scale than fMRI allows us to image, or because the experience is not comparable across species or paradigms is a topic for future research. Notably, neuroimaging does not allow for causal inferences, and current findings may not generalize to other fear-conditioning protocols; thus, our data primarily highlight the challenges of translational research. Acknowledging that we may currently lack the tools to translate knowledge about the microscopic organization of deep brain structures in nonhuman animals to mesoscale functioning in humans seems preferable over forcefully drawing parallels where this is not justified, or necessary.
